# Associations of DDX60L With the Clinical Features and Prognosis of Hepatocellular Carcinoma

**DOI:** 10.3389/fonc.2022.761021

**Published:** 2022-02-11

**Authors:** Ziqi Ye, Xin Zhang, Yanfang Zhang, Linqing Liu, Zixue Xuan, Ping Huang

**Affiliations:** ^1^Department of Clinical Pharmacy, The First Affiliated Hospital, Zhejiang University School of Medicine, Hangzhou, China; ^2^Laboratory Medicine Center, Department of Pathology, Zhejiang Provincial People’s Hospital, Affiliated People’s Hospital, Hangzhou Medical College, Hangzhou, China; ^3^International Medical Department, The First Affiliated Hospital of University of Science and Technology of China, Division of Life Sciences and Medicine, University of Science and Technology of China, Hefei, China; ^4^Clinical Pharmacy Center, Department of Pharmacy, Zhejiang Provincial People’s Hospital, Affiliated People’s Hospital, Hangzhou Medical College, Hangzhou, China; ^5^Key Laboratory of Endocrine Gland Diseases of Zhejiang Province, Zhejiang Provincial People’s Hospital, Affiliated People’s Hospital, Hangzhou Medical College, Hangzhou, China

**Keywords:** hepatocellular carcinoma, DDX60L, prognosis, HCV infection, HCC

## Abstract

**Objective:**

Although the pathogenesis of hepatocellular carcinoma (HCC) is still unclear, hepatitis C virus (HCV) infection is considered a common cause of HCC. It has been reported that DDX60L can inhibit HCV replication, but its role in HCC is still poorly understood.

**Methods:**

The expression levels of DDX60L in HCC tissues and in tissues adjacent to the tumor and their correlation with the clinicopathological features of patients were analyzed. We also used Kaplan–Meier curves of overall survival (OS) with Cox regression analysis and log-rank test to investigate the prognostic value of DDX60L in HCC. We further performed cell proliferation, Transwell, and wound healing assays to elucidate the role of DDX60L in HCC using the siRNA-DDX60L Hep3B or HCCLM3 cell line.

**Results:**

Univariate analysis showed that sex, Edmondson grade, microvascular invasion, tumor stage (III–IV/I–II), AFP, and DDX60L expression were strongly associated with the prognosis of HCC patients. The results of multivariate analysis further suggested that DDX60L might be an independent prognostic factor for OS in patients with HCC (*P*_moderate/low_ = 0.015, *P*_high/low_ = 0.011). The low DDX60L expression in HCC patients with no-metastasis, age ≥55 years, tumor size <5 cm, Edmondson grade = I–II, microvascular invasion, no cirrhosis, HBV positivity, tumor stage = III–IV, AFP >20 μg/L, and multiple tumor was associated with poorer prognosis (*P <*0.05). Moreover, the expression of DDX60L was significantly lower in HCC samples (N = 285) than in the normal tissues adjacent to the tumor (N = 167, *P <*0.001). There were no HCV-related HCC patients in this study. Additionally, we found that DDX60L knockdown can promote the proliferation of Hep3B cells, migration and invasion ability of Hep3B and HCCLM3 cells.

**Conclusion:**

We found that the downregulation of DDX60L expression correlated with poor prognosis in patients with HCC, which may be independent of the HCV-related pathway. Furthermore, DDX60L significantly inhibited the proliferation of Hep3B cells, migration and invasion of Hep3B and HCCLM3 cells. Therefore, DDX60L can serve as a prognostic biomarker and therapeutic target for HCC.

## Introduction

Hepatocellular carcinoma (HCC) is the most common type of liver cancer and the fourth leading cause of cancer-related deaths worldwide, with 45.3% of HCC patients found in China (1). Additionally, HCC is an intractable tumor caused by multiple factors and shows the participation of multiple genes; the lack of specific and early diagnostic markers leads to a large proportion of patients showing advanced HCC during their diagnosis (2). Systemic therapy, namely, sorafenib, lenvatinib, regorafenib, ramucirumab, and cabozantinib have been recommended by the NCCN guidelines to treat patients with advanced HCC (3). Pembrolizumab is recommended for patients with advanced HCC who have received prior treatment with sorafenib (4). Despite these advances, the prognosis of HCC remains poor (5), and novel biomarkers and therapeutic targets need to be developed.

Viral infections, namely, hepatitis B virus (HBV) and/or hepatitis C virus (HCV), are specific risk factors for HCC development (6–8). In the United States, HCV-related HCC patients are common (1). Direct-acting antivirals (DAAs) may improve sustained virologic responses in patients with HCV (9, 10), which ultimately reduces the incidence of HCC (11–13). HCV induces an effective interferon (IFN) response early in the host infection, leading to interferon-stimulated gene (ISG) expression as the first line of defense against viral infection (14, 15). The DEAD-box helicase (DDX) family contains some ISG products that contribute to antiviral defense by sensing and fighting viral infections (16). In general, the DDX family has conserved structural domains and functions and is involved in the majority of RNA metabolic processes, from transcription to degradation (17). It has been shown that the DDX family plays an important role in tumor development. For example, high expression of DDX1 is present in neuroblastoma and retinoblastoma and promotes tumor progression, with effects on RNA transcription, export, and translation (18). The upregulated expression of DDX17 was closely associated with poor prognosis in HCC, and DDX17 knockout could inhibit the invasive capacity of HCC cells, indicating that DDX17 is a potential prognostic marker for HCC (19). Dead box polypeptide 60-like (DDX60L) is a member of the DDX family (20), and it was found that DDX60L is highly expressed in pancreatic ductal adenocarcinoma (PDAC) cells and that DDX60L knockdown significantly induced apoptosis and reduced the metastasis of PDAC cells (21). However, little is known about the correlation between DDX60L and the clinicopathological features of HCC. Therefore, in this study, we aimed to investigate the role of DDX60L and its clinical relevance in HCC.

## Materials and Methods

### Tissue Microarray and Cell Culture

HCC samples were obtained from the Zhejiang Provincial People’s Hospital (Hangzhou, China), and written informed consents were obtained from all patients. This study was approved by the Ethics Committee of the Zhejiang Provincial People’s Hospital. The tissue microarrays contained 285 HCC tissue samples and 167 tumor-adjacent normal tissue samples. The follow-up period was >5 years. Overall survival (OS) was calculated from the date of surgery to the end date of the follow-up or the date of death. The Hep3B cell line was purchased from the Hunan Fenghui Biological Technology Co., Ltd. HCCLM3 were obtained from the Zhongshan Hospital, Fudan University, Shanghai, China. Hep3B and HCCLM3 cells were cultured in DMEM with 10% FBS, 100 μg/ml penicillin, and 0.1 mg/ml streptomycin at 37°C and 5% CO_2_ in a humidified incubator.

### Immunohistochemical Staining

Immunohistochemical staining was performed according to the standard methods. Briefly, 5 µm sections from the tissue microarrays were baked at 70°C for 2 h. Sections were then deparaffinized in xylene, rehydrated using an ethanol concentration gradient, and heated in a pressure cooker for 3 min in an antigen retrieval citra solution (pH 8.0, catalog number ZLI-9072, Zhongshan Golden Bridge Biotechnology) for antigen repair, and sealed with 3% hydrogen peroxide for 15 min to inhibit the activity of endogenous peroxidase. Subsequently, tissue microarray slices were incubated for 16 h at 4°C with the primary antibody against DDX60L (dilution, 1:400; catalog number bs-14236R; Beijing Biosynthesis Biotechnology Co., Ltd.), followed by incubation with EnVision+ System-HRP (catalog number K4001, Dako). Finally, the staining was visualized with the DAB Substrate kit (catalog number DA1010-2, Solarbio) and counterstained with hematoxylin (22).

### Scoring of Immunohistochemical Staining

The pathologist scored the immunohistochemical staining of DDX60L based on the proportion and intensity of the positively stained cells. The staining intensity was assessed using a four-grade grading system: 3 (strong), 2 (moderate), 1 (weak), and 0 (negative). The percentage of DDX60L-positive cells was scored as follows: 0 (less than 5%), 1 (6–25%), 2 (26–50%), 3 (51–75%), and 4 (>75%) (22). The staining intensity scores and the percentage of positive cells were multiplied to obtain the final immunohistochemical staining score of DDX60L. Score ranges of 0–3, 4–8, and 9–12 were used to define low, moderate, and high expression levels of DDX60L, respectively.

### Small Interfering RNA (siRNA) Transfection

Hep3B and HCCLM3 cells were transfected with a mixture of siRNA (100 nM) and Lipofectamine 2000 reagent (Guangzhou Ruibo Biological Technology Co., Ltd.) according to the manufacturer’s protocol. The siRNA sequences used were as follows: siRNA-DDX60L-1, sense: GGUAUUCCAGCAUAUUAAAdTdT, antisense: UUUAAUAUGCUGGAAUACCdTdT; and siRNA-DDX60L-2, sense: GAUUCAGAGUGCAUAUAAAdTdT, antisense: UUUAUAUGCACUCUGAAUCdTdT. Cells were collected 24 or 48 h after transfection for subsequent experiments. Moreover, the downregulation of DDX60L expression was confirmed *via* real-time PCR.

### Cell Proliferation Assay

Briefly, 200 μl of Hep3B cells or siRNA-DDX60L Hep3B cells, and HCCLM3 cells or siRNA-DDX60L HCCLM3 cells, were seeded into 96-well cell culture-treated plates, and cultured for 12, 24, or 48 h. Then cell proliferation assay was performed using Cell Counting Kit-8 (CCK-8; Hangzhou Fude Biological Technology Co., Ltd.) according to the manufacturer’s protocol (23).

### Transwell Assay and Wound Healing Assay

A total of 200 μl of Hep3B cells or siRNA-DDX60L Hep3B cells (2 × 10^4^ cells/well), and also HCCLM3 cells or siRNA-DDX60L HCCLM3 cells, were seeded into the upper chamber of a Transwell (Corning, USA). DMEM supplemented with 10% FBS (600 μl) was added to the lower chamber and incubated at 37°C for 24 h. Cell migration was analyzed using methanol and 0.3% crystal violet staining.

Hep3B or siRNA-DDX60L Hep3B cells, and also HCCLM3 cells or siRNA-DDX60L HCCLM3 cells, were seeded at a density of 2 × 10^5^ cells/cm^2^. Scratch wounds were made using a 10 µl pipette tip. Cells were washed twice with PBS and then cultured in serum-free DMEM at 37°C for 48 h. An inverted light microscope was used to observe the cells, and the ImageJ software (version 1.52) was used to analyze the effect of DDX60L on the migration ability of Hep3B or HCCLM3 cells.

### Statistical Analysis

We used the IBM Statistical Package for Social Sciences (SPSS) software (version 20.0; SPSS Inc., Chicago, IL, USA) to perform the statistical analyses. Because the DDX60L expression was divided into low-, moderate-, and high-level expression groups, and we considered it as an ordinal categorical variable, the Mann–Whitney U test was applied separately to evaluate the difference in DDX60L expression in clinicopathological parameters. We used the Kaplan–Meier curve to compare the OS. Univariate and multivariate Cox regression models were used to analyze the independent risk factors associated with the OS. The mean and SD of at least three separate cell experiments were obtained. Unpaired t-tests were used to compare the differences between the two groups. Statistical significance was set at *P <*0.05.

## Results

### Clinical Characteristics and Prognosis of Patients With HCC

The clinical characteristics of HCC patients are shown in **Table 1**, with no significant differences except for the Edmondson grade (I–II/III–IV), microvascular invasion, tumor stage, and AFP (*P* = 0.007, 0.015, 0.006, and 0.015, respectively). We used a univariate Cox proportional model to analyze the correlation between the clinicopathological characteristics and OS in 285 patients with HCC. Furthermore, no history of HCV infection was found in any of the patients. As shown in **Table 2**, sex (female/male), Edmondson grade (III–IV/I–II), microvascular invasion (±), tumor stage (III–IV/I–II), AFP (>20/≤20), and DDX60L expression (moderate/low, and high/low) were significantly associated with OS (*P <*0.05), while age (≥55/<55), tumor size (≥5/<5), metastasis (M1/M0), HBV antigen (±), multiple tumor (±), and cirrhosis (±) were not significantly associated with OS (*P >*0.05). In addition, multivariate Cox regression models showed that DDX60L expression (moderate/low: HR = 0.248, 95% CI: 0.080–0.765, *P* = 0.015; high/low: HR = 0.153, 95% CI: 0.036–0.647, *P* = 0.011) was independent prognostic factor for HCC.

**Table 1:** Clinical characteristics of HCC patients.

Clinical parameters	Patients (N = 285)	No. of events	HR (95% CI)	*P*-value
**Age, years**			1.508 (0.854–2.664)	0.156
<55	110	29 (26.4%)		
≥55	172	33 (19.2%)		
**Sex**			0.541 (0.276–1.061)	0.071
Male	232	46 (19.8%)		
Female	51	16 (31.4%)		
**Size, cm**			0.618 (0.349–1.096)	0.098
<5	149	27 (18.1%)		
≥5	129	34 (26.4%)		
**Edmondson grade**			0.396 (0.201–0.783)	**0.007**
I–II	203	39 (19.2%)		
III–IV	48	18 (37.5%)		
**Microvascular invasion**			0.438 (0.223–0.860)	**0.015**
Absence	109	18 (16.5%)		
Presence	90	28 (31.1%)		
**Metastasis**			0.330 (0.118–0.927)	0.060
M0	269	55 (20.4%)		
M1	16	7 (43.8%)		
**Tumor stage**			0.454 (0.255–0.807)	**0.006**
I–II	187	32 (17.1%)		
III–IV	96	30 (31.3%)		
**Cirrhosis**			0.819 (0.450–1.489)	0.512
Absence	102	20 (19.6%)		
Presence	183	42 (23.0%)		
**HBV antigen**			1.037 (0.517–2.079)	0.918
Negative	57	13 (22.8%)		
Positive	221	49 (22.2%)		
**HCV antigen**			–	–
Negative	285	–		
Positive	0			
**AFP, μg/L**			0.475 (0.258–0.873)	**0.015**
≤20	125	19 (15.2%)		
>20	146	40 (27.4%)		
**Multiple tumor**			0.642 (0.313–1.314)	0.223
Yes	45	13 (28.9%)		
No	237	49 (20.7%)		

HR, hazard ratio; HBV, hepatitis B virus; HCV, hepatitis C virus; Bold number means P < 0.05.

**Table 2:** Univariate and multivariate Cox regression analyses for the clinicopathological parameters in HCC patients.

Clinical parameters	Category	Univariate analysis			Multivariate analysis		
		HR	95% CI	*P*-value	HR	95% CI	*P*-value
Age (years)	≥55/<55	0.690	0.418–1.136	0.145	–	–	–
Sex	Female/male	1.784	1.009–3.154	**0.047**	1.831	0.884–3.793	0.104
Tumor size (cm)	≥5/<5	1.546	0.932–2.565	0.092	–	–	–
Edmondson grade	III–IV/I–II	1.980	1.132–3.462	**0.017**	1.644	0.788–3.427	0.185
Metastasis	M1/M0	2.065	0.936–4.553	0.072	–	–	–
Microvascular invasion	±	2.284	1.262–4.136	**0.006**	1.457	0.314–6.760	0.631
Tumor stage	III–IV/I–II	2.059	1.251–3.390	**0.004**	1.546	0.334–7.154	0.578
HBV antigen	±	1.052	0.570–1.940	0.872	–	–	–
Cirrhosis	±	1.143	0.671–1.947	0.623	–	–	–
AFP (μg/L)	>20/≤20	1.992	1.153–3.441	**0.013**	0.975	0.477–1.994	0.945
Multiple tumor	±	1.377	0.747–2.540	0.306	–	–	–
DDX60L expression	Moderate/Low	0.386	0.165–0.900	**0.028**	0.248	0.080–0.765	**0.015**
	High/Low	0.216	0.070–0.671	**0.008**	0.153	0.036–0.647	**0.011**

HR, hazard ratio; HBV, hepatitis B virus; Bold number means P < 0.05.

### DDX60L Expression Correlates With the Overall Survival and Clinicopathological Characteristics of Patients With HCC

**Figure 1:**
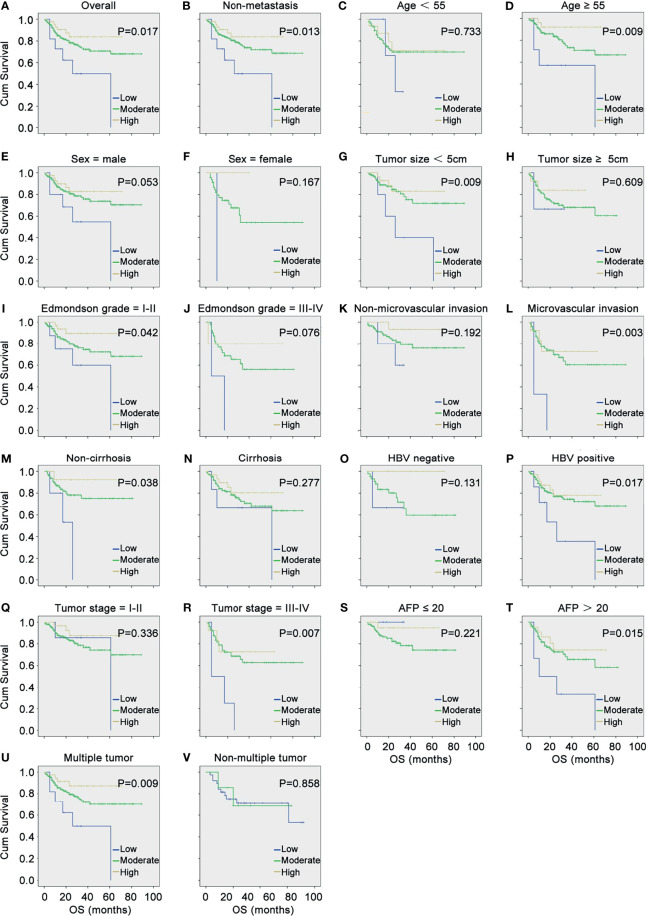
DDX60L expression correlated with the overall survival (OS) and clinicopathological features of HCC. **(A)** Kaplan–Meier survival analysis and log-rank test were used to compare the OS among different DDX60L expression groups; Kaplan–Meier survival analysis and log-rank test were used to analyze the correlation between DDX60L expression and OS stratified by non-metastasis **(B)**; age **(C, D)**; sex **(E, F)**; tumor size **(G, H)**; Edmondson grade **(I, J)**; microvascular invasion **(K, L)**; cirrhosis **(M, N)**; HBV **(O, P)**; tumor stage **(Q, R)**; AFP **(S, T)**; and multiple tumor **(U, V)**.

Subsequently, the correlation between DDX60L expression and OS was assessed using Kaplan-Meier survival analysis and log-rank test. As shown in **Figure 1A**, HCC patients with low levels of DDX60L expression had a shorter OS (*P* = 0.017). In a stratified analysis (**Figures 1B–V**), the downregulation of DDX60L expression correlated with poor prognosis in HCC patients with non-metastasis (*P* = 0.013), age ≥55 (*P* = 0.009), tumor size <5 cm (*P* = 0.009), Edmondson grade = I–II (*P* = 0.042), microvascular invasion (*P* = 0.003), non-cirrhosis (*P* = 0.038), HBV positive (*P* = 0.017), tumor stages III–IV (*P* = 0.007), AFP >20 μg/L (*P* = 0.015), and multiple tumor (*P* = 0.009).

### Differential Expression of DDX60L in HCC Tissues and Normal Tissues Adjacent to Tumors

**Figure 2:**
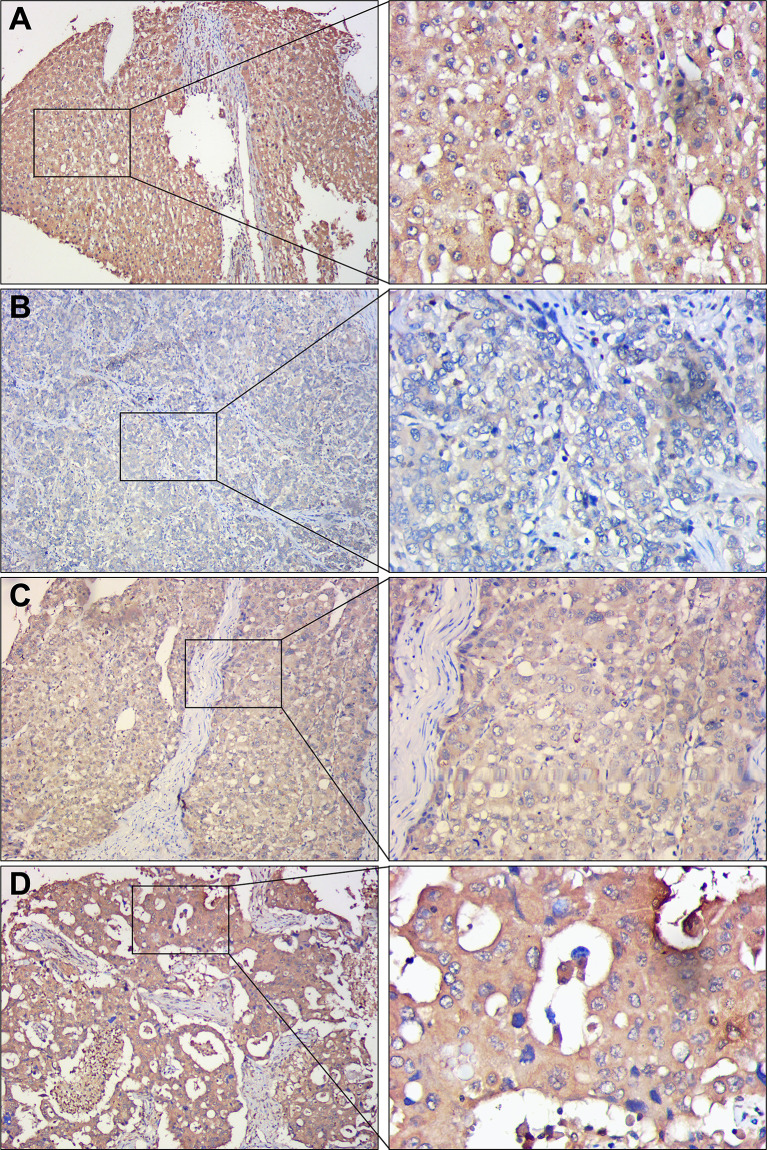
Representative immunohistochemical staining of DDX60L in HCC tissues and tumor-adjacent normal tissues. Magnification in the left column, ×100; magnification in the right column, ×400. **(A)** tumor-adjacent normal tissues, showing high DDX60L expression in cell membrane and cytoplasm; **(B)** Hepatocellular carcinoma tissues, showing a low DDX60L expression in the cell membrane and cytoplasm; **(C)** Hepatocellular carcinoma tissues, showing moderate DDX60L expression in cell membrane and cytoplasm; **(D)** Hepatocellular carcinoma tissues, showing high DDX60L expression in the cell membrane and cytoplasm.

**Figure 3:**
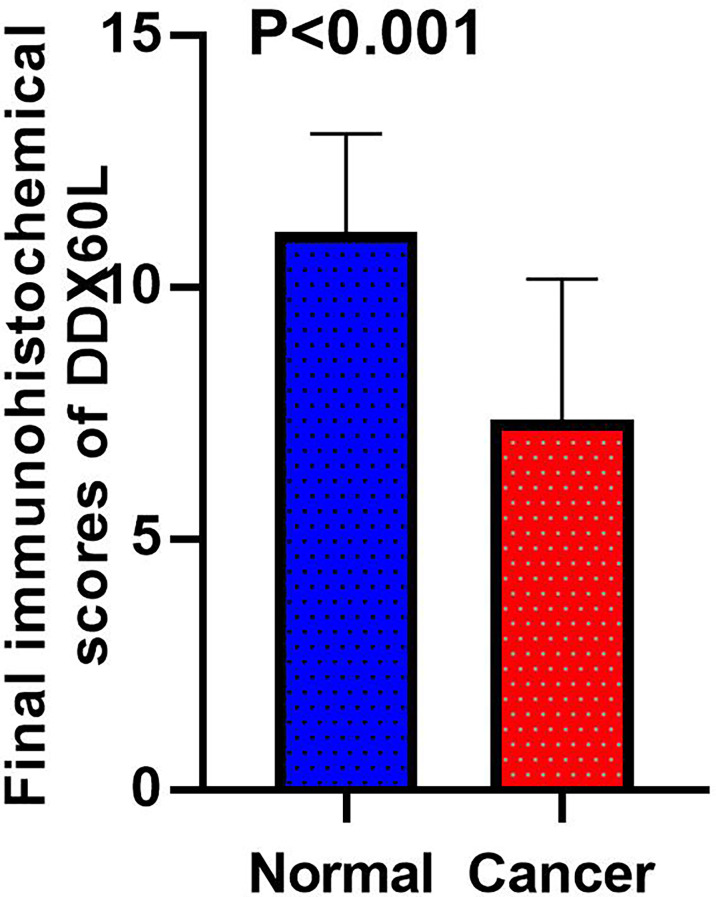
Final immunohistochemical scores of DDX60L in HCC tissues (N = 285) and tumor-adjacent normal tissues (N = 167).

To assess the differences in DDX60L expression between HCC and tumor-adjacent normal tissues, we compared the final immunohistochemical scores of DDX60L in 285 HCC tissues and 167 tumor-adjacent normal tissues. Immunohistochemical results showed that DDX60L expression was significantly decreased in HCC tissues compared with that in adjacent normal tissues (**Figure 2**). As shown in **Figure 3**, the final immunohistochemical scores of DDX60L were significantly reduced in HCC (7.37 ± 2.79, N = 285) compared with normal tissue adjacent to the tumor (11.10 ± 1.96, N = 167, *P <*0.001). To explore the clinical significance of DDX60L expression, we further analyzed the correlation between the expression of DDX60L and different clinicopathological features of HCC patients. As shown in **Table 3**, DDX60L expression was significantly lower in patients with a tumor size ≥5 cm (N = 129) than in patients with a tumor size <5 cm (N = 149, *P* = 0.018). This suggests that tumor size is significantly associated with DDX60L expression in patients with HCC.

**Table 3:** Correlation of DDX60L expression and clinicopathological variables.

Clinical parameters	Patients (N = 285)	DDX60L expression, N			Z	*P*-value
		Low	Moderate	High		
**Age, years**					−0.380	0.704
<55	110	4	90	16		
≥55	172	7	136	29		
**Sex**					−1.577	0.115
Male	232	10	180	42		
Female	51	1	47	3		
**Size, cm**					−2.365	**0.018**
<5	149	5	113	31		
≥5	129	6	110	13		
**Edmondson grade**					−1.110	0.267
I–II	203	8	159	36		
III–IV	48	2	41	5		
**Microvascular invasion**					−0.163	0.870
Absence	109	5	86	18		
Presence	90	3	74	13		
**Metastasis**					−1.229	0.219
M0	269	11	213	45		
M1	16	0	16	0		
**Tumor stage**					0.616	0.735
I–II	187	7	148	32		
III–IV	96	4	79	13		
**Cirrhosis**					−0.903	0.367
Negative	102	5	83	14		
Positive	183	6	146	31		
**HBV antigen**					−0.054	0.957
Negative	57	3	44	10		
Positive	221	7	180	34		
**AFP, μg/L**					0.651	0.722
≤20	125	4	98	23		
>20	146	6	118	22		
**Multiple tumor**					2.218	0.330
Yes	45	0	38	7		
No	237	11	188	38		

DDX60L expression was divided into low, moderate and high expression groups based on immunohistochemistry scores of 0–3, 4–8, and 9–12, respectively. HBV, hepatitis B virus; N, number of patients; Bold number means P < 0.05.

### DDX60L Inhibits the Proliferation, Invasion, and Migration of HCC Cells *In Vitro*

**Figure 4:**
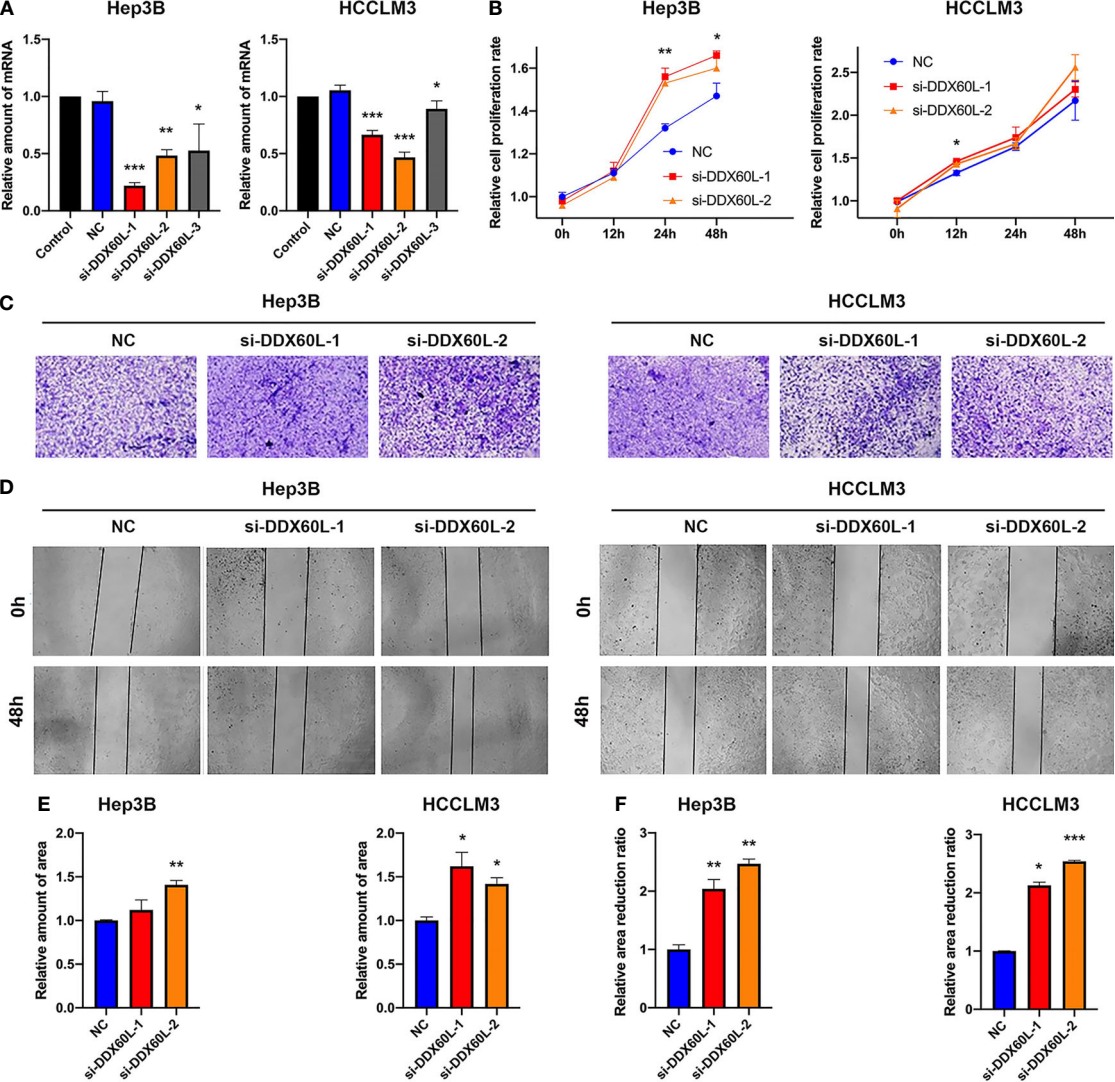
DDX60L inhibited the proliferation of Hep3B, the invasion and migration of Hep3B and HCCLM3 cells. **(A)** si-DDX60L-1 and si-DDX60L-2 had good inhibitory effects; **(B)** The results of CCK-8 assays showed that the DDX60L knockdown could promote the proliferation of Hep3B cells, but hardly affected the proliferation of HCCLM3 cells; **(C, E)** The results of Transwell assays showed that DDX60L knockdown could promote the invasion of Hep3B and HCCLM3 cells. Magnification, ×10; **(D, F)** Wound healing assays demonstrated that DDX60L knockdown could increase the migration of Hep3B and HCCLM3 cells. Magnification, ×10. *P < 0.05 and **P < 0.01, compared with NC. NC, negative control. ***P < 0.001

We found that si-DDX60L-1 and si-DDX60L-2 had a good inhibitory effect in Hep3B and HCCLM3 cells (**Figure 4A**). The results of the cell proliferation assay showed that the downregulation of DDX60L expression promoted the proliferation of Hep3B cells, but had little effect on proliferation of HCCLM3 cells (**Figure 4B**). Subsequently, we found that DDX60L knockdown could promote the invasion ability of Hep3B and HCCLM3 cells (**Figures 4C, E**). Similar to results of Transwell assay, we also found that DDX60L knockdown significantly increased the migration of Hep3B and HCCLM3 cells (**Figures 4D, F**). Overall, these findings suggest that DDX60L significantly inhibited the proliferation of Hep3B cells, migration and invasion of Hep3B and HCCLM3 cells *in vitro*.

## Discussion

Prior studies have noted the importance of the DDX family in HCC (24). For example, DDX3 is deregulated in HCV-associated HCC and plays an important role in cell growth (24); DDX5 suppressed HCC by inducing autophagy (25), and there was a reduced expression of DDX5 in HBV-related HCC patients, who had a poor prognosis (26). Additionally, DDX5 inhibits HBV biosynthesis and Wnt signaling in HBV-related HCC (27). Oncogene DDX17 is a potential prognostic marker for HCC (19), while DDX20 inhibited HCC by regulating the CDC42-integrin pathway (28). As a member of the DDX family, the functions of DDX60L in HCC are still unknown. There is evidence that DDX60L may inhibit viral RNA replication (29). It is well known that HCV infection is related to the prognosis of HCC, because HCV infection induced cirrhosis, and then contribute to the increased incidence of HCC (30, 31).

In the present study, we found that the expression of DDX60L was strongly associated with poor OS, suggesting that DDX60L could be an independent risk factor for HCC. Additionally, the results of functional studies showed that DDX60L knockdown significantly promoted the proliferation of Hep3B cells, migration and invasion of Hep3B and HCCLM3 cells. Nevertheless, very little is currently known about whether DDX60L inhibits HCC progression by only depending on its role in the HCV infection pathway. Thus, we tried to explore the influence of HCV on the role of DDX60L, and found DDX60L suppressed the development of HCC independent of the HCV-related pathway, because there were no HCV-related HCC patients in this study. Furthermore, recent studies suggest the DDX family was one of the typical splicing regulators that played a central role in RNA metabolism and usually functions as part of the spliceosome (32). A comprehensive analysis showed that tumor samples had 30% more AS events than normal samples (33), and abnormal AS was strongly associated with invasion, metastasis, poor prognosis of tumors (34). DDX60L is one of the important members of the DDX family, so we speculate that it may play a role in HCC by regulating AS, but the conclusion deserves further confirmation in the future.

To the best of our knowledge, this is the first study about the role of DDX60L in HCC, which will be very helpful to understand other molecular mechanisms of HCC. However, there are some limitations. For instance, the relationship between disease-free survival (DFS) and DDX60L, the influence of sorafenib treatment and alcohol intake on the role of DDX60L, the specific regulatory mechanism of DDX60L in HCC, have not been elucidated. Therefore, further studies need to be carried out to address these limitations and clarify the specific mechanism of DDX60L in HCC.

## Conclusion

In summary, we observed the downregulation of DDX60L is closely associated with poor prognosis of HCC patients, and DDX60L may be involved in HCC independent of the HCV-related pathway. Then, we found DDX60L inhibited the proliferation, migration, and invasion of HCC cells. Taken together, our findings might highlight the significance of DDX60L in the tumorigenesis, development and clinical outcome of HCC, and suggest that DDX60L can serve as a prognostic biomarker and therapeutic target for HCC.

## Data Availability Statement

The original contributions presented in the study are included in the article/supplementary material. Further inquiries can be directed to the corresponding authors.

## Ethics Statement

The studies involving human participants were reviewed and approved by the Ethics Committee of Zhejiang Provincial People’s Hospital. The patients/participants provided their written informed consent to participate in this study.

## Author Contributions

ZY and ZX designed this study. XZ and LL collected the data of the patients and performed the experiment of immunohistochemical staining. YZ and ZY contributed to the data analysis. ZY, ZX and PH participated in the writing and revision of the manuscript. All authors listed have made a substantial, direct, and intellectual contribution to the work and approved it for publication.

## Funding

This study was supported by the Natural Science Foundation of Zhejiang Province (Grant Number LYY21H310008) and the Fundamental Research Funds for the Central Universities (Grant Number WK9110000183).

## Conflict of Interest

The authors declare that the research was conducted in the absence of any commercial or financial relationships that could be construed as a potential conflict of interest.

## Publisher’s Note

All claims expressed in this article are solely those of the authors and do not necessarily represent those of their affiliated organizations, or those of the publisher, the editors and the reviewers. Any product that may be evaluated in this article, or claim that may be made by its manufacturer, is not guaranteed or endorsed by the publisher.
